# Review of Natural Compounds for Potential Skin Cancer Treatment

**DOI:** 10.3390/molecules190811679

**Published:** 2014-08-06

**Authors:** Tawona N. Chinembiri, Lissinda H. du Plessis, Minja Gerber, Josias H. Hamman, Jeanetta du Plessis

**Affiliations:** Centre of Excellence for Pharmaceutical Sciences, North-West University, Private Bag X6001, Potchefstroom 2520, South Africa; E-Mails: Tawona.Chinembiri@nwu.ac.za (T.N.C.); Lissinda.DuPlessis@nwu.ac.za (L.H.P.); Minja.Gerber@nwu.ac.za (M.G.); Sias.Hamman@nwu.ac.za (J.H.H.)

**Keywords:** anti-cancer, melanoma, plant, natural, dietary, phytochemical

## Abstract

Most anti-cancer drugs are derived from natural resources such as marine, microbial and botanical sources. Cutaneous malignant melanoma is the most aggressive form of skin cancer, with a high mortality rate. Various treatments for malignant melanoma are available, but due to the development of multi-drug resistance, current or emerging chemotherapies have a relatively low success rates. This emphasizes the importance of discovering new compounds that are both safe and effective against melanoma. *In vitro* testing of melanoma cell lines and murine melanoma models offers the opportunity for identifying mechanisms of action of plant derived compounds and extracts. Common anti-melanoma effects of natural compounds include potentiating apoptosis, inhibiting cell proliferation and inhibiting metastasis. There are different mechanisms and pathways responsible for anti-melanoma actions of medicinal compounds such as promotion of caspase activity, inhibition of angiogenesis and inhibition of the effects of tumor promoting proteins such as PI3-K, Bcl-2, STAT3 and MMPs. This review thus aims at providing an overview of anti-cancer compounds, derived from natural sources, that are currently used in cancer chemotherapies, or that have been reported to show anti-melanoma, or anti-skin cancer activities. Phytochemicals that are discussed in this review include flavonoids, carotenoids, terpenoids, vitamins, sulforaphane, some polyphenols and crude plant extracts.

## 1. Introduction

Cancer is considered as a major cause of mortality worldwide, while the incidence of skin cancer is ever increasing in countries where such tumors are prevalent. Between 1970 and 2007, among all of the documented cancers in Canada, melanoma had the second highest increase in mortality rate [1] and it is estimated that skin cancer is the most common form of cancer in the United States of America (USA). According to Erb *et al.* [2], skin cancers are the most frequently diagnosed malignancies in Caucasians worldwide, whilst their incidence keeps increasing, due to increased exposure to ultra-violet (UV) radiation. Cancer of the skin is characterized by an imbalance towards too little apoptosis, or too much cell proliferation and survival in the epidermis [3]. Although UV radiation is the leading cause of skin cancer, other causative agents include viruses, mutagens in food, mutagens in chemicals and genetic susceptibility [4,5]. Skin cancer can be prevented by controlling, or eliminating these causative agents. Skin cancer can be effectively removed by hindering blood supply to the tumor (anti-angiogenesis), which curbs tumor growth and enhances patient survival. Most cancer cells develop ways to evade apoptosis, or exhibit defective apoptosis mechanisms, thus allowing uncontrollable cell development [2]. The apoptosis process is therefore the major target of anti-cancer chemotherapeutics. Currently, skin cancer is treated by surgical removal, radiation therapy, chemotherapy, or cryosurgery, amongst other techniques. Both 5-fluorouracil and imiquimod are used in topical chemotherapies of superficial, basal cell carcinoma (BCC) and squamous cell carcinoma (SCC) *in situ*, while only imiquimod is approved for topical therapy of cutaneous malignant melanoma (CMM) [6,7,8]. The different treatment methods have both their advantages and disadvantages, therefore, choosing a treatment option is never easy and the preferred choice is influenced by factors, such as the site of the cancer, health status of the patient, as well as patient and doctor opinions.

The main problems that exist with chemotherapeutic agents are severe adverse effects and multi-drug resistance formation. Some of the methods by which cancer cells become resistant to therapies are drug efflux systems, amplification of drug targets, or changes in drug kinetics [9,10,11]. Various strategies have been attempted to overcome drug resistance, such as the use of nanoparticles, liposomes and micellar drug delivery vehicles, with some reported successes [11]. The adverse effects of cancer chemotherapy can be treated symptomatically, but in some instances such secondary treatments may be very toxic, which is unacceptable to some cancer patients [12,13,14].

**Figure 1:**
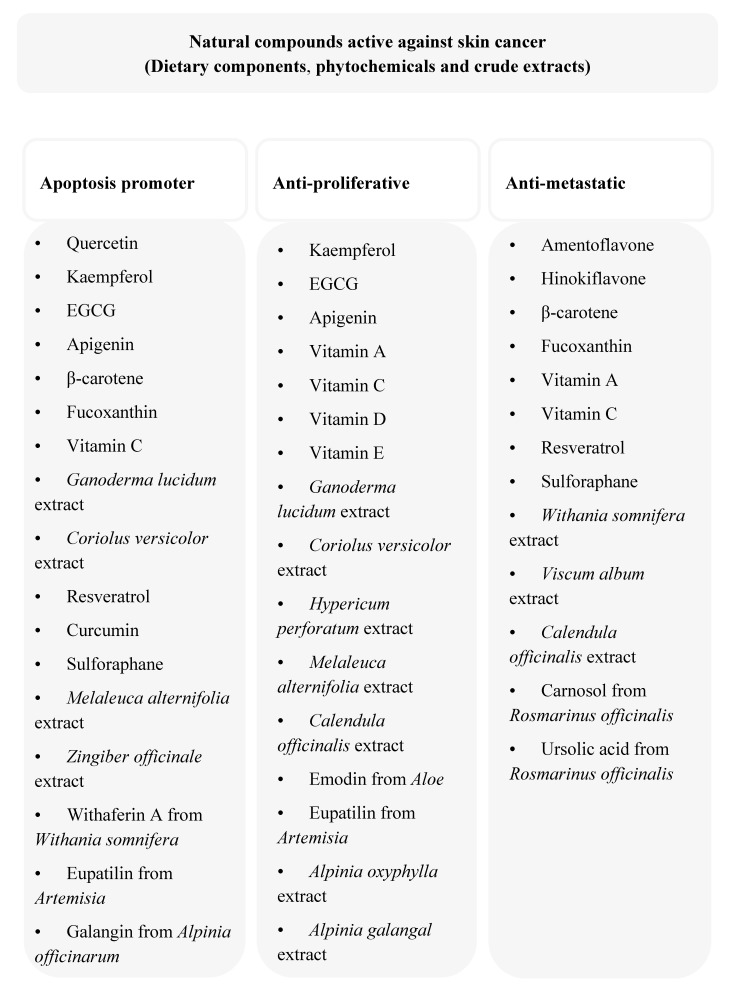
A scheme showing the anti-melanoma actions of the compounds and extracts discussed in this review.

There has been a growing interest in the use of complementary and alternative medicines (CAM), due to the disadvantages associated with conventional cancer chemotherapies and the supposed advantages of more natural treatment options [15]. Phytochemical compounds from extracts of plant roots, bulbs, barks, leaves, stems and others have shown promising potential as anti-cancer drugs, or for serving as lead compounds in the synthesis of new drugs. They are often utilized as traditional medicines in the form of home-made tinctures, teas, or crude extracts. Disadvantages of natural products and traditional medicines include variation in preparation methods and therefore also chemical composition, dosage determination and adjustment, and the suitable route of administration. Although much research on compounds of natural origin to produce new drug substances occurs, research, specifically aimed at naturally derived medicines to optimize dosages for the intended route of administration and to design the most effective dosage forms, has become essential [16]. The worldwide increase in the use of CAM is mainly due to the false perception, or belief that natural products are safe, while scientifically proven information on clinical aspects of some CAMs generally does not exist [15,17]. Figure 1 shows an overview of the anti-melanoma natural products that are discussed in this review.

## 2. Natural Sources of Anti-Cancer Compounds

An abundance of natural resources for medicinal use exist worldwide, of which many have not yet been exploited for possible application in the pharmaceutical industry. Over 50% of all available drugs on the market originated from natural sources, of which over 70% of anti-cancer agents have their origin in natural sources. Natural sources include plants, animals, microbes and marine life [17]. Plants are the most utilized natural resource for applications in the pharmaceutical science and still comprise the leading natural source for new drugs and lead compounds, due to their accessibility and abundance. To date, only a few naturally derived drugs exist on the market that target skin related cancers, whereas none have yet been approved for topical application. This could be attributed to the known side effects of these agents when topically applied to the skin.

The following sections offer an overview of compounds from different natural sources that have been found to exhibit activity against different types of cancer, with a specific focus on melanoma.

### 2.1. Marine Sources

In recent years, interest in the potential of marine fauna and flora as a source of novel medicinal agents has grown significantly. Substantive research, aimed at utilizing this vast natural resource, is being carried out worldwide. The high anti-tumor potency of agents, discovered from marine resources, reflects the high potential of the ocean as a possible source of anti-cancer drugs [17]. Extracts from sponges, algae and marine cyanobacteria have shown strong anti-cancer activities [18,19,20]. Laminarans, fucoidans, alginic acids and carrageenans are some of the compounds isolated from marine sources that have been found to exhibit effective anti-cancer activities. An assortment of polysaccharides from marine animals, bacteria and fungi have also been tested for anti-cancer activity, of which some were found promising for further drug development [18]. Although various anti-cancer compounds from marine origin have been isolated and tested *in vitro* and *in vivo* and taken through different stages of clinical testing, only four anti-cancer drugs of marine origin have reached the market so far. These anti-cancer drugs are cytarabine, trabectedin, eribulin mesylate and brentuximab vedotin, derived from *Cryptotethia crypta*, *Ecteinascidia turbinate*, *Halichondria okadai* and *Symploca hydnoides*, respectively [21,22].

Cytarabine is a pro-apoptotic compound that also acts by inhibiting cell growth in cancerous cells. In 1998, the Food and Drug Administration (FDA) had approved the first marine derived compound, cytarabine, for clinical use as an anti-cancer agent in the treatment of acute myelogenous leukemia. Trabectedin, a derivative of Caribbean tunicate, was next approved for treatment of metastatic soft tissue carcinoma in 2007 by the European Commission. In 2009, trabectedin received even further approval for the treatment of relapsed, platinum sensitive ovarian cancer. Eribulin mesylate was then approved by the FDA for clinical use as part of a third line treatment regimen for advanced, metastatic breast cancer in 2010 [22]. Brentuximab vedotin received FDA approval for treatment of systemic, anaplastic, large cell lymphoma and Hodgkin’s lymphoma in 2011. These four anti-cancer drugs have been further subjected to the various stages of clinical trials for their possible use in more diverse types of cancer, either alone or as part of a treatment regimen.

Aplidin, bryostatin-1, salinosporamide and zalypsis are other examples of marine-derived compounds that are currently undergoing clinical trials for potential use as anti-cancer drugs [16]. Many more marine derived compounds with anti-cancer potential are currently undergoing pre-clinical investigation [20,23].

### 2.2. Microbial Sources

The tumor regression activity of bacteria was discovered and used clinically over a century ago, when Coley [24] observed that tumors in patients that had been accidentally infected with *Streptococcus pyogenes* had degenerated. Such regression was due to an immune response stimulated by the bacterial infection and it was this discovery that caused the advent of cancer immunotherapy. Ever since, much research has been performed on microbes to explore their anti-neoplastic potential. The chemical diversity and ease of access of microbes with respect to collection, culturing and fermentation make them an extremely relevant source of pharmaceutically active compounds [17]. Anthracyclins, bleomycins, staurosporins and actinomycins are groups of microbially derived anti-cancer compounds in clinical use [16,17].

Whole bacteria can be used in their live, attenuated, or genetically modified forms to stimulate immune responses, but this may potentially result in side effects that can be avoided by using bacterially derived products instead. Ongoing research is carried out on the use of bacterial toxins and spores and on the use of bacteria as vectors for gene therapy. Toxins from microorganisms can have advantageous effects in humans, such as destroying rapidly dividing cells in tumors [25].

### 2.3. Plant Sources

Over 50% of all drugs currently in clinical use worldwide have originated from compounds extracted from plants [26]. From 1960 to 1982, the National Cancer Institute (NCI) in the USA embarked on a plant collection program, aimed at boosting progress in the discovery of plant derived anti-cancer agents [27]. During this time, a wide range of cytotoxic agents were discovered from plant extracts, but very few of these managed to reach the market for clinical use. The development of taxanes and camptothecins as drugs for clinical use took over twenty years [17,27,28].

The vinca alkaloids, including vincristine, vinblastine and vinorelbine, were the first plant-derived anti-cancer agents to gain approval for clinical use. Thereafter came the discovery and approval of the podophyllotoxin derivatives (*i.e.*, etoposide and teniposide), taxanes (*i.e.*, paclitaxel and docetaxel) and camptothecin derivatives (*i.e.*, irinotecan and topotecan) [27,29]. The mechanism of action of the vinca alkaloids involves interaction with tubulin so as to disrupt the assembly of the mitotic spindle, which in turn leads to the demise of actively dividing cells [29]. Contrary to the vinca alkaloids, taxanes work by stabilizing the microtubule, instead of destabilizing it. The stabilization of the microtubule results in an imbalance between tubulin and microtubules, which affects normal cellular function and in turn results in cell death. Camptothecins and podophyllotoxins inhibit topoisomerase I through different mechanisms, but both cause disruption of the cell division process [17].

An example of a plant that is currently being investigated for possible use in the treatment of advanced pancreatic cancer, non-small cell lung cancer, metastatic colorectal cancer and breast cancer, is *Viscum album L*. It was found that the combination of *Viscum album L*. whole extract and gemcitabine had been relatively well tolerated [30]. A phase II clinical trial is reported to have been conducted on using a green tea extract, Polyphenon E, for the treatment of chronic lymphocytic leukemia. Shanafelt *et al.* [31] found that this green tea extract had been relatively effective and well tolerated in patients.

Berberine, a naturally occurring isoquinolone alkaloid was tested in combination with doxorubicin on human melanoma cells and *in vivo* on mice. It was found that this combination had suppressed tumor growth *in vitro* and *in vivo* [32]. Extracts of *Tilia amurensis* and *Camellia sinensis* were tested on cancer cell lines originating from the skin and they were found to have cytotoxic effects *in vitro* [33,34]. It is further reported that some phytochemicals, such as epigallocatechin-3-gallate and apigenin have demonstrated a higher inclination for cytotoxicity towards melanoma and epidermoid carcinoma cells, compared to normal cells and such chemicals are increasingly being investigated [35,36].

## 3. Anti-Cancer Dietary Components and Phytochemicals

Phytochemicals having anti-inflammatory, immuno-modulatory and anti-oxidant properties, generally have the highest potential of exhibiting chemo-preventive behavior in skin cancers [37]. Numerous attempts have been made to find the correlation between antioxidant properties of phytochemicals and their anti-cancer potential. Although no concrete evidence of such a correlation has been found yet, the anti-oxidant activity of a phytochemical is being regarded as an indication of potential anti-cancer activity [38,39]. Carotenoids, flavonoids and terpenoids are some of the groups of phytochemicals with high anti-cancer potential [40,41,42].

### 3.1. Flavonoids

**Figure 2:**
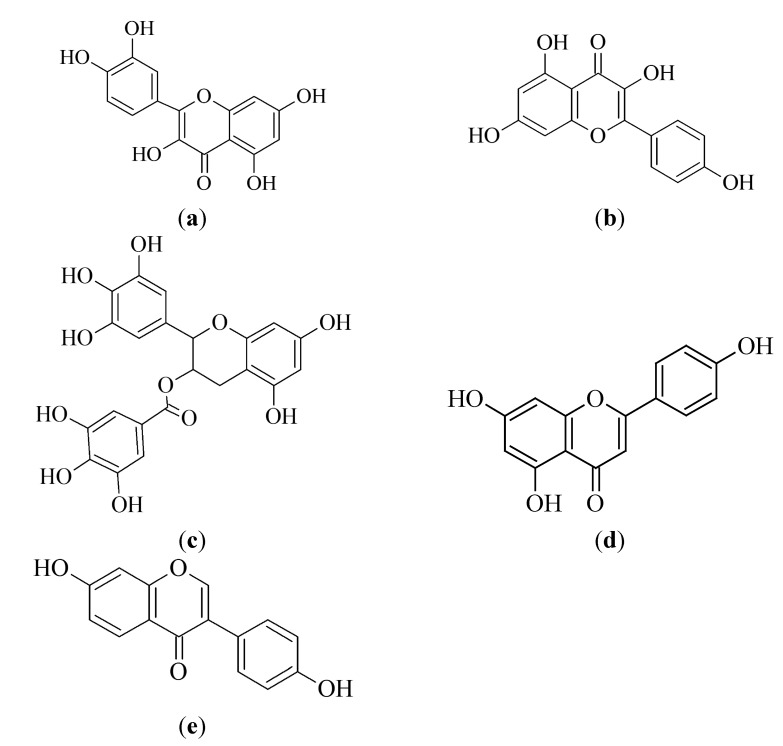
Chemical structures of selected flavonoids possessing anti-cancer potential: (**a**) quercetin, (**b**) kaempferol, (**c**) EGCG, (**d**) apigenin and (**e**) daidzein.

Flavonoids are acetogenins from plant and flower pigments [43] and their chemical structures are characterized by two benzene rings that are connected through a linear carbon chain and an aromatic chromophore [44]. The bright colors of plant parts rich in flavonoids are due to the aromatic chromophore. The main groups of flavonoids include flavonols, flavanones, flavones, isoflavones, flavan-3-ols (catechins) and anthocyanins. Figure 2 illustrates the chemical structures of some flavonoids that have been found to exhibit anti-cancer activities and are discussed in the sections that follow.

Flavonoids are well known for their anti-oxidant (or free radical scavenging) and chelating properties and are continuously being investigated for application in the treatment of diseases [45]. The anti-oxidant activity of flavonoids can act as both a trigger of tumorigenesis and/or as an inhibitor of tumorigenesis, depending on other physiological factors. Not all flavonoids would therefore be useful in cancer chemotherapy or chemo-prevention [38,39]. Some flavonoids have further demonstrated to absorb ultra-violet B (UVB) rays, hence contributing to their photoprotective effect in plants, by behaving as UV filters and protecting underlying elements [46]. This photoprotective property of flavonoids has been adapted and investigated in human cells and in mice models, to determine whether flavonoids and their derivatives could be used as photoprotective agents in humans [47].

#### 3.1.1. Quercetin

Quercetin is a flavonol that is identified by the presence of hydroxyl groups on positions 3, 5, 7, 3' and 4' of the flavonol skeleton [48] [Figure 2a]. This flavonol is insoluble in cold water, slightly soluble in hot water and soluble in alcohol [48]. Quercetin seems to be one of the most effective flavonoids with respect to its biological activities [49]. The anti-cancer activity of quercetin is mainly attributed to its anti-oxidant and anti-inflammatory properties [50]. Research must, however, continue to determine the actual pathways through which quercetin exhibits its anti-cancer activity [51,52].

Quercetin is the most abundant flavonol in the human diet and it is found in plants in many glycosidic forms, such as galactosides, rhamnosides, arabinosides, or glucosides [53,54]. The main sources of quercetin include, but are not limited to, apples (*Malus domestica*), tomatoes (*Solanum lycoperscium*), tea (*Camellia sinensis*), grapes (*Vitis vinifera*), Ginkgo (*Ginkgo biloba*) and St John’s Wort (*Hypericum perforatum*) [48]. Onions (*Allium cepa*), although generally ingested in small amounts, are also known to contain large amounts of quercetin [48,53]. Other sources are dark chocolate (*Theobroma cacao*), capers (*Capparis spinosa*), cloves (*Syzygium aromaticum*), black elderberries (*Sambucus nigra*) and oregano (*Origanum vulgare*) [55].

Since quercetin is found in many food substances, research has been conducted to determine whether these food substances offer any protective properties against cancer formation and its progression. To date, a number of different synergistic and antagonistic effects have been found through which quercetin acts biologically to contribute towards the strength of its chemo-protective and anti-metastatic actions, or lack thereof [56]. Evidence indicated that quercetin acts against melanoma by affecting cell viability at low concentrations and by inducing apoptosis at higher concentrations [57]. During a study by Zhang *et al.* [58], quercetin induced apoptosis in murine melanoma cells (B16–BL6) by attenuating the expression of B cell lymphoma 2 (Bcl-2) and potentiating caspase-3 activity. Another mechanism through which quercetin acts according to the findings by Olson *et al.* [59], is one where quercetin potentiates the c-fos gene expression induced by UVB, while at the same time inhibiting phosphoinositide-3 kinase (PI3-K), leading to both potentiation and inhibition of carcinogenesis. Most recently, quercetin has been reported to block UVB induced oxidative stress and deoxyribonucleic acid (DNA) damage, which in turn induces apoptosis in mouse epidermal cells. The same study showed that quercetin inhibited the generation of reactive oxygen species (ROS) and restored the expression of anti-oxidant enzymes in the C141 mouse epidermal cells [60]. Cao *et al.* [61] recently reported that the anti-melanoma activities of quercetin may be due to inhibitory effects on *signal transducer and activator of transcription 3* (STAT3), an oncogenic protein.

Nonionic and anionic topical formulations, containing quercetin, were applied to the dorsal skin of UVB irradiated hairless mice. The nonionic formulation was lipid rich, while the anionic formulation had low lipid content and contained the anionic hydrophilic colloid, Carboxypolymethylene-Carbopol^®^ 940. The *in vivo* results indicated that both formulations had resulted in the inhibition of an increase in myeloperoxidase activity and a decrease in glutathione, both associated with UVB irradiation skin damage [62]. Glycosides and polymethoxylate derivatives of quercetin have been identified as good candidates for use in the topical delivery of quercetin, as some of them have exhibited improved therapeutic indices, metabolic stability and anti-inflammatory activity *in vitro* [63]. Nanoparticle formulations and micro-emulsions containing quercetin have worked well for targeting drug delivery to the skin *in vitro* [64,65].

#### 3.1.2. Kaempferol

Kaempferol is one of the most abundant flavonoids found in a wide variety of food components, hence the interest in its medicinal properties. Kaempferol is a poorly water soluble flavonoid, found in tea (*Camellia sinensis*), strawberries (*Fragaria ananassa*), green chilli (*Capsicum frutescens*), carrot (*Daucus carota*), pumpkin (*Cucurbita pepo*), brinjal (*Solanum melongena*), broccoli (*Brassica oleracea var. italica*), propolis, grapefruit (*Citrus paradisi*), apples (*Malus domestica*), beans (*Phaseolus vulgaris*) and onions (*Allium cepa*) [44,66,67,68]. The chemical structure of kaempferol is illustrated in Figure 2b. Epidemiological studies have shown a positive relationship between consumption of foods with high kaempferol content and a reduction in the incidence of cancer (lung, ovarian, gastric and pancreatic) and cardiovascular diseases [69].

There are a number of possible mechanisms through which kaempferol exerts its anti-cancer effects, such as promoting apoptosis and inhibiting cell proliferation [67]. Kaempferol has been found to block choroidal melanoma cell cycle progression in the G2/M phase [70]. Chao *et al.* [71] had developed submicron emulsion systems for the transdermal delivery of kaempferol and found that the use of an appropriate vehicle could significantly influence the flux, the deposition amount in skin and the lag time. In a skin permeation study conducted by Park *et al.* [72], kaempferol in solution (1,3-butylene glycol-ethanol) was able to permeate the skin barrier of albino mice. An investigation by Park *et al.* [72] regarding the anti-oxidant and cellular membrane protective effects of kaempferol also generated encouraging results.

#### 3.1.3. Epigallocatechin-3-gallate

Epigallocatechin-3-gallate [EGCG, chemical structure shown in Figure 2c] is a stable and water soluble member of the group of flavonoids referred to as flavan-3-ols [73]. Flavan-3-ols are mainly found in tea (black, green, oolong) (*Camellia sinensis*), red wine (from *Vitis vinifera*), strawberry (*Fragaria ananassa*) and cocao (*Theobroma cacao*) products, with green tea being the main source [40,44]. Geographical data indicates the probability that the incidence of prostate cancer is lower in certain Japanese and Chinese populations, due to their high green tea consumption [74].

EGCG is capable of inducing apoptosis and cell cycle arrest in melanoma cells (A374 and Hs-294T), alone and in combination with vorinostat *in vitro* [75,76]*.* Treatment with a combination of EGCG and interferon has also shown synergistic anti-proliferative effects against human melanoma cells *in vitro* and against a mice melanoma model *in vivo* [77]. The mechanisms through which EGCG exerts these effects include down regulation of apoptosis inhibiting proteins, or cell survival promoting proteins (Bcl-2, D1 and cyclin dependent kinase 2 (cdk2), the up regulation of Bcl-2 associated X protein (Bax), a pro-apoptosis protein, the activation of caspases-3, -7 and -9, and through the induction of tumor suppressor proteins (p16^INK4a^, p21^WAF1/CIP1^ and p2^KP1^) [36,78]. Other suggested mechanisms include inhibiting the activation of the epidermal growth factor receptor and the downstream adapter protein Shc in human skin carcinoma cells (A431) [79]. Nandakumar *et al.* [80] investigated the anti-skin cancer effects of EGCG, focusing particularly on its effects on silenced tumor suppressor genes. Treatment of human skin carcinoma cells (A431) with EGCG had resulted in a reduction of DNA methylation, which in turn led to protein expression of the DNA hypermethylation-silenced tumor suppressor genes, p*16^INK4a^* and CDK interacting protein 1/p21 (*Cip1/p21*). EGCG, however had no effect on the expression of the tumor suppressor genes in normal epidermal cells [80]. These results for EGCG were confirmed by the outcomes of another study on A431 cells, namely inhibition of proliferation and the induction of apoptosis in human skin cancer cells, but through inactivation of β catenin signaling [34]. During a study by Nihal *et al.* [36], EGCG showed pro-apoptotic activity, selective towards melanoma cells and not towards the normal melanocytes. Lu *et al.* [73] very recently reported compelling evidence regarding the induction of DNA damage and high genetic mutation frequency in normal lung and skin cells by high concentrations of EGCG, which could possibly cause cancer.

Besides the possible use as an anti-carcinogenic agent, EGCG is considered a good candidate for use as a photoprotectant. Skin photoprotection is an important aspect of skin cancer prevention and studies have illustrated that EGCG shows high potential to prevent the skin from photo induced damage, the leading cause of skin cancer. Sevin *et al.* [81] found that the topical application of EGCG to rat skin, thirty minutes prior to UVA exposure, had reduced the formation of sunburn cells.

#### 3.1.4. Apigenin

Apigenin is a flavone, chemically referred to as 4',5,7,-trihydroxyflavone [Figure 2d], which forms yellow, needle like crystals in its pure form and is commonly found in celery (*Apium graveolens*), oranges (*Citrus sinensis*), tea (*Camellia sinensis*), parsley (*Petroselinum crispum*), thyme (*Thymus vulgaris*) and onions (*Allium cepa*) [82]. Apigenin is one of the most bioactive flavones in plants and epidemiologic observations have shown that flavone rich diets are associated with a reduction in the risk of developing certain cancers [83]. Such observations have stimulated research on the anti-cancer activities of apigenin.

The anti-cancer activities of apigenin have been observed *in vitro* in various cell lines, such as head and neck squamous cell carcinoma cells, melanoma cells and liver cells. The mechanisms of action, as determined by Chan *et al.* [35], include inducing cell cycle arrest in the G_2_/M phase, up regulating tumor necrosis factor receptor (TNF-R) and the TNF related apoptosis inducing ligand receptor (TRAIL-R) apoptotic pathway, down regulating Bcl-2, and activating caspase-3. The combinations of all these actions result in the chemo-protective effects of apigenin. Apigenin has been found to exhibit UVB radiation protective effects on human keratinocytes *in vitro* and on mice skin tissue *in vivo*, by interfering with cell survival and cell proliferation via the nuclear factor kappa-light-chain-enhancer of activated B cells (NF-κB) and mitogen activated protein kinase (MAPK) pathways [84]. Furthermore, an oncogenic kinase (Src) is inhibited by apigenin, resulting in an inhibition of UVB induced expression of cyclooxygenase 2 (COX-2), thus reducing the inflammatory and oncogenic effects associated with COX-2 [85].

Ethosomes have been formulated together with apigenin and it was determined that apigenin loaded ethosomes resulted in a higher skin deposition of apigenin, compared to liposomes *in vitro* and *in vivo* [86]. The influence of nano-encapsulation of apigenin was investigated by Das, *et al.* [87], who found that the encapsulated apigenin could be a more ideal formulation, compared to the free apigenin, as the encapsulated apigenin was able to penetrate the nucleus and in turn result in higher apoptotic effects. Topical application of nano-encapsulated apigenin resulted in the inhibition of tumorigenesis in UVB exposed, Swiss, albino mice. However, a combination of oral and topical apigenin intake proved a more potent inhibitor of carcinogenesis in these mice [88].

#### 3.1.5. Daidzein

Daidzein is a soy isoflavone [chemical structure shown in Figure 2e], which is highly soluble in alkaline environments and is part of a group of compounds, called phytoestrogens [89]. It has demonstrated some chemo-protective potential in the skin, since topical application of daidzein in a study resulted in effective photo-protection [90]. *In vitro* studies showed that daidzein was able to inhibit UVB induced production of hydrogen peroxide within cells and therefore the protection of the keratinocytes. Daidzein and genistein have been investigated as synergistic cytotoxic agents in various studies and evidence showed that the two isoflavones had worked well together [89,91,92]. Franz cell based, *in vitro* diffusion studies and tape-stripping showed that minimal amounts of daidzein had managed to penetrate through the skin [89]. This unfavorable skin permeation characteristic of daidzein may be the reason for the limited research that has been carried out with regards to its potential use as a topical photo- and chemo-protectant.

#### 3.1.6. Biflavonoids

In recent years there has been an increased interest in the medicinal properties of biflavonoids. Biflavonoids are dimers of flavones, flavonols and flavanones [93,94], and they are known to inhibit melanogenesis [95]. Compounds that fall in this group include amentoflavone, podocarpusflavone, volkensiflavone, fukugetin, hinokiflavone. Some biflavonoids whose anti-melanoma activity has been investigated and reported are amentoflavone and hinokiflavone. Guruvayoorappan and Kuttan [96], reported that amentoflavone (from *Biophytum sensitivum*) inhibits metastasis of B16F10 melanoma cells *in vivo* by inhibiting tumor invasion, migration, proliferation and angiogenesis. These effects were found to be possibly linked to attenuation of the effects of matrix metalloproteinases (MMPs), vascular endothelial growth factor (VEGF) and extracellular signal-regulated kinase (ERK). Amentoflavone has also been found to inhibit endothelial cell migration and angiogenesis which are linked to placental growth factor 1 (PlGF-1) [97]. In addition to these actions amentoflavone has been reported to prolong survival of metastatic tumor-bearing mice [98]. The potential MMP-9 inhibitory action of hinokiflavone was investigated using pharmacophore modeling and it was reported to have inhibitory effects on MMP-9 which in turn may result in anti-metastasis effects [99].

### 3.2. Carotenoids

**Figure 3:**
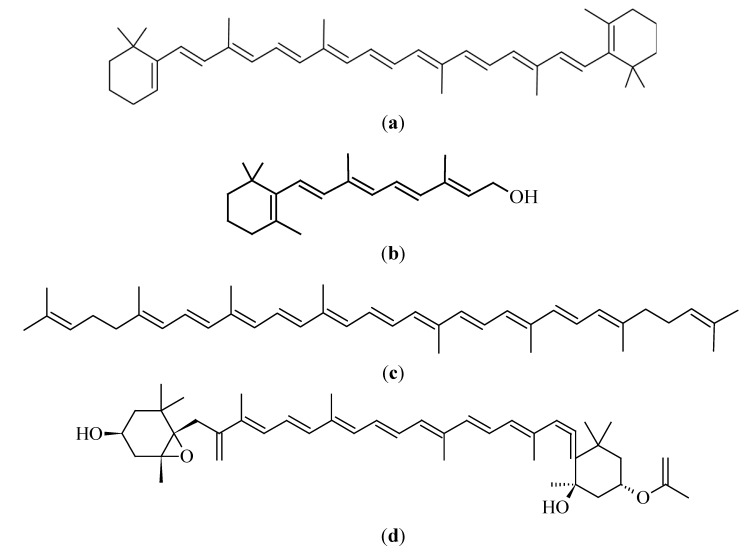
Chemical structures of selected carotenoids: (**a**) β-carotene, (**b**) retinol, (**c**) lycopene and (**d**) fucoxanthin.

Carotenoids are fat-soluble pigments that are commonly found in nature, especially in plants [100]. These compounds are made up of eight C_5_ isoprenoids that are combined to form C_40_ tetraterpenoids, with various chemical modifications (e.g., hydrogenation, isomerization, dehydrogenation, presence of oxygen functions, *etc.*) to form different carotenoids. The distinctive conjugated double bond system of carotenoid structures acts as a light absorbing chromophore, which gives the yellow, orange or red colors to tomatoes, dark green vegetables, oranges and other bright colored food substances [101]. Figure 3a–d illustrate the chemical structures of carotenoids, discussed in this section. Carotenoids are divided into two main groups, namely hydrocarbons, which are highly fat soluble carotenes, and xanthophylls, which are relatively polar carotenoids that contain oxygen [102]. Pro-vitamin A carotenoids are carotenoids with an unsubstituted β ring, for example β-carotene. Pro-vitamin A carotenoids act as precursors of retinol in the body [103]. There are more than 500 carotenoids in nature, but the commonly studied carotenoids include β-carotene, α-carotene, lycopene, lutein, astaxanthin, fucoxanthin and canthaxanthin [42,104].

Much research has been undertaken with respect to the medicinal properties of carotenoids and they are believed to be potential agents for preventing cancer, diabetes and cardiovascular diseases [100]. The medicinal properties of carotenoids are assumed to be due to their anti-oxidant activities that reduce DNA damage by free radicals after exposure to UV light, but other mechanisms are also under investigation. Pro-vitamin A carotenoids are converted into retinol and exert the effects of retinol within the body, which is essential in proliferation, maintenance and differentiation of cells within the epithelia [105].

#### 3.2.1. β-Carotene

The most widely studied carotenoid and one of the most abundant carotenoids in the human diet is β-carotene. Some of the dietary sources of β-carotene are carrots (*Daucus carota*), spinach (*Spinacia oleracea*), kale (*Brassica oleracea* var*. acephala*), pepper (*Capsicum spp.*), pumpkin (*Cucurbita pepo*), sweet potatoes (*Ipomoea batatas*) and cantaloupe (*Cucumis melo* var*. cantalupensis*) [104]. Both melanocytes and keratinocytes are able to accumulate and store β-carotene for conversion into retinol when required [106].

β-Carotene is able to induce apoptosis in melanoma cells *in vitro* by activating caspases-3, -8 and -9 via a caspase cascade [107]. According to Guruvayoorappan and Kuttan [108], the mechanism of anti-neoplastic activity of β-carotene in murine melanoma cells may include the regulation of Bcl-2, p53 and caspase-3, which then stimulates apoptosis. In another study by Guruvayoorappan and Kuttan [109], the effect of β-carotene on tumor specific angiogenesis, which affects tumor growth, was explored. In this study, it was found that β-carotene may have inhibited tumor specific angiogenesis by inhibiting the activation or nuclear translocation of various transcription factors [109]. β-Carotene seemed to have activated Bax, a pro-apoptosis protein, in melanoma cells, although no evidence of apoptosis actually occurring in the cells have been found. In some instances, inhibition or up regulation of gene expression does not always translate into a particular protein function, as there may be other processes that could overcome the effects of the inhibition, or up regulation, of which this is one such example [110].

Initially, β-carotene was regarded in high esteem as a chemo-protectant, due to promising *in vitro* results, but a number of controlled studies in human subjects and the murine model have shown ambiguous and conflicting results with respect to the anti-cancer effects of β-carotene. It was shown that a diet high in carotenoids (β-carotene included) may be related to a decreased risk for melanoma [111,112]. In a case study, metastatic melanoma regressed in a patient after she had changed to a diet that was rich in anti-oxidants (β-carotene included). This may have been a case of spontaneous regression, but the possibility also existed that it had been as a result of her change in diet [113]. In another case, oral supplementation of a combination of β-carotene with other anti-oxidants (*i.e.*, vitamin C, vitamin E, selenium and zinc) resulted in a significant increase in the incidence of melanoma in women, but not in men [114]. This effect may have been as a result of a synergistic effect of the supplement components, or due to one particular compound. The incidence of melanoma declined after the anti-oxidant supplementation was stopped, thus supporting the idea that anti-oxidants are not necessarily beneficial to the treatment, or prevention of skin cancers [115]. Prior to these studies, it had been established that β-carotene levels in blood had no underlying influence on the incidence of melanoma [116,117]. Twelve year supplementation with β-carotene in men had shown no influence, positive or negative, on the incidence of malignant neoplasms, melanoma included [116]. Such findings have made it difficult to ascertain whether β-carotene actually is beneficial *in vivo*.

#### 3.2.2. Lycopene

Lycopene is one of the most common acyclic carotenoids and it is the pigment found in red and orange-fleshed fruits and vegetables. Examples are watermelon (*Citrullus lanatus*), papaya (*Carica papaya*), tomato (*Solanum lycopersicum*), guava (*Psidium guajava*), grapefruit (*Citrus paradisi*), apricot (*Prunus armeniaca*) and peaches (*Prunus persica*) [101,104]. Lycopene [Figure 3b] is an unsubstituted hydrocarbon, making it a highly lipophilic carotenoid with no vitamin A activity.

As with β-carotene, lycopene blood levels of 30.9–40.8 μg/dL on average have demonstrated no influence on the incidence of melanoma [117]. The risk of melanoma in subjects with high serum levels of lycopene is not significantly different from the risk in those with middle or low levels [118]. This further illustrated that anti-cancerous effects of compounds are usually selective for a particular type of cancer, as some evidence suggests that lycopene has cancer preventative actions in models of lung, colon, liver and mammary gland carcinogenesis [102]. Lycopene acts by trapping platelet-derived growth factor-BB, which in turn inhibits migration and the signaling of fibroblasts that are induced by melanoma cells. This effect, however, does not result in a reduction in cell viability or metastatic potential, once again revealing that there are various processes at play within the complex physiology of cancer [119,120].

#### 3.2.3. Fucoxanthin

Fucoxanthin is a brown or orange marine derived carotenoid, with its main sources including edible brown seaweeds, brown algae and heterokants [104]. Examples of the sources of fucoxanthin are *Undari pinnatifida, Hijika fusiformis*, *Laminaria japonica*, *Sargassum fulvellum* [121] and *Fucus evanescens* [122].

Reduced incidences of tumors, anti-proliferation, cell cycle arrest, apoptosis induction and inhibition of metastasis are some of the anti-cancer effects that are exerted by fucoxanthin [123]. Similar to β-carotene, fucoxanthin seems to induce apoptosis via the caspase pathways [124]. Fucoxanthin inhibits growth of SK-MEL-28 and B16F10 melanoma cells in a concentration dependent manner [122,124]. A recent study has shown that fucoxanthin suppresses the metastatic potential of murine melanoma cells, by down regulating some proteins involved in cell interaction, cell migration and cell adhesion, *i.e.*, MMP 9, CD44 and C-X-C chemokine receptor 4 (CXCR4) [125]. Topical application of fucoxanthin in UV irradiated guinea-pigs resulted in the suppression of tyrosinase activity and of the melanogenesis process in melanoma. Melanogenesis suppression was achieved through inhibition of messenger ribonucleic acid (mRNA) expression of COX-2 and through the inhibition of a number of receptors (*i.e.*, melanocortin 1, prostaglandin E, endothelin A and p75 neurotrophin) [126]. Intra-peritoneal injection of fucoxanthin has been shown to inhibit the *in vivo* growth of intra-dermal B16F10 tumors in mice [124].

### 3.3. Vitamins

**Figure 4:**
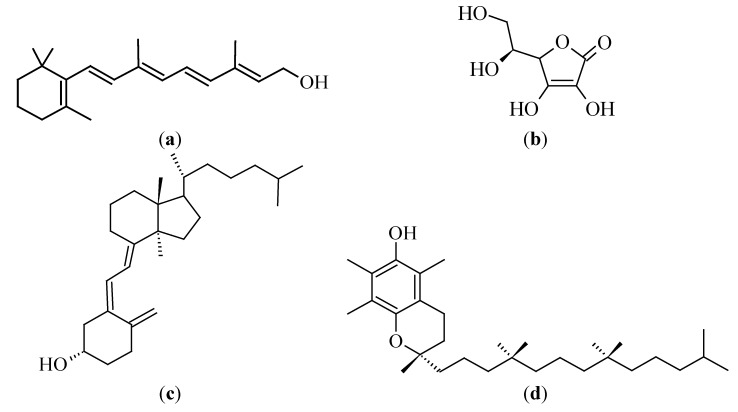
Chemical structures of (**a**) vitamin A, (**b**) vitamin C, (**c**) vitamin D3 and (**d**) vitamin E (α-tocopherol).

Vitamins are families of essential compounds that cannot be produced within the human body and are therefore taken in with food and as supplements. The importance of vitamins lies within the various roles they play in physiological processes. In this section reference is made to vitamins A, C, D and E, as illustrated in Figure 4.

#### 3.3.1. Vitamin A (Retinol)

Vitamin A (or retinol) is well known for its roles in eye physiology, reproduction, the immune system and bone development. The main sources of retinol of importance are eggs, milk, cheese, meat and liver [127]. Retinoids are known for being key players in the processes of differentiation and cell proliferation, as they possess anti-proliferative and pro-differentiating activities [128].

Epidemiological studies have been conducted to determine whether serum levels of retinol would have any influence on the incidence of melanoma. These studies have shown that there was no direct relationship between retinol levels and the propensity to develop melanoma [117,129]. In a large cohort study conducted, the intake of retinol supplements was found to be related to a reduced risk for developing melanoma, especially in women [130]. The conflicting findings of the different studies may have been due to for example not having fully considered other risk factors, and due to possible bias in the selection of candidates. Another possible explanation could have been that some retinoic acid receptors (RAR-β2) are silenced in human melanoma and the retinol is unable to exert its full anti-proliferative effects in melanoma [128]. Another controversy, related to vitamin A, is that retinyl palmitate, the storage form of retinol in the skin and used in sunscreens, has been found to possess photo-carcinogenic potential, thus raising questions about its safety for topical applications. Retinyl palmitate has been in use for decades and no observations have been made with respect to it increasing an individual’s susceptibility to develop skin cancer, hence supporting the notion that it is relatively safe for topical use [131,132].

The pharmacological effects of vitamin A are not only ascribed to retinol itself, but also to its metabolites, namely the retinoic acids. Studies regarding the anti-cancer activity of retinol sometimes include retinoic acid. *In vitro* and *in vivo* testing, aimed at determining the mechanisms of action of retinol, have found that retinol mainly acts by inhibiting angiogenesis. The anti-angiogenesis action of retinol was observed in a murine melanoma angiogenesis model. Mice that had been treated with 13-*cis*-retinoic acid for five days showed a 50% decrease in the number of tumor directed capillaries. The anti-angiogenesis effect of 13-*cis*-retinoic acid seemed to have been as a result of inhibition of VEGF production, the inhibition of the migration of endothelial cells and the inhibition of tube formation [133]. Meyskens and Fuller [134] found that 13-*cis*-retinoic acid, retinol and β-all-*trans*-retinoic acid were able to inhibit proliferation in human melanoma cells *in vitro*, which fueled further studies on the use of retinol and related agents as possible anti-cancer compounds in melanoma. A recent study conducted by Ribeiro, *et al.* [135] showed that a combination of all-*trans-*retinoic acid and endoxifen had a high anti-proliferative and anti-migratory effect on melanoma cells, but demonstrated no toxicity towards normal endothelial cells. It has also been reported that the combination of all-*trans-*retinoic acid and EGCG had a synergistic anti-cancer effect on melanoma, as all-*trans*-retinoic acid up regulates the 67-kDa Laminin receptor, which is thought to be crucial to the action of EGCG [127].

#### 3.3.2. Vitamin C (Ascorbic Acid)

Epidemiological studies that have investigated the possible relationship between vitamin C intake and the risk of developing melanoma, had shown no benefits of vitamin C in preventing melanoma [136]. It was found that patients who had consumed food with high vitamin C content, especially orange juice, had in fact demonstrated a higher likelihood of developing melanoma [129,136]. However, *in vitro* and *in vivo* efficacy studies have proven otherwise. The main sources of vitamin C are citrus fruits, broccoli (*Brassica oleracea var. italica*), green pepper (*Capsicum annuum*), tomatoes (*Solanum lycoperscium*), strawberries (*Fragaria ananassa*) and melons.

Vitamin C appears to act by inducing apoptosis and by inhibiting cell proliferation and cell growth. Apoptosis induction by vitamin C is thought to occur by way of pro-oxidant activities that can be blocked by N-acetyl-L-cysteine, a potent anti-oxidant. Vitamin C is generally known to have anti-oxidant activity, but contrary to expectations, its anti-cancer activity in melanoma cells has proven to be related to oxidative stress instead, due to the caspase 8 pathway [137]. According to research conducted by Neena, *et al.* [138], low concentrations of ascorbate resulted in concentration dependent melanoma cell death, but as the ascorbate concentrations were increased, a proliferative effect was observed. Ascorbic acid has been found to up regulate levels of p53 and p21 tumor suppressor proteins, resulting in cell cycle arrest in the G1 phase [139]. Another proposed mechanism for the anti-cancer activity of vitamin C includes angiogenesis. It is thought that vitamin C suppresses the expression of vascular endothelial growth factor (VEGF) in melanoma cells, thus enabling it to suppress angiogenic processes, which could result in tumor regression [140]. Type 1 insulin like growth factor receptor (IGF-1R) and COX-2 expression are also down regulated by vitamin C, resulting in anti-proliferative effects, as observed by Seung Koo, *et al.* [141].

#### 3.3.3. Vitamin D

Vitamin D is a fat soluble vitamin that is mainly derived from 7-dehydrocholesterol, which is converted into pre-vitamin D_3_ in the skin, due to sun exposure [142]. Salmon, mackerel, bluefish, cod liver oil, mushrooms, egg yolks and yeasts are reputably good dietary sources of vitamin D_3_ [143]. Vitamin D exists as ergocalciferol, cholecalciferol, calcidiol and calcitriol [144]. The overall importance of vitamin D lies in its key roles in the immune system, bone development and cell proliferation [142].

Epidemiological studies have shown no relationship between vitamin D intake, nor pre-diagnostic vitamin D serum levels and a susceptibility to develop melanoma [145,146]. A direct relationship between plasma levels of vitamin D and the risk of non-melanoma skin cancers in women has, however, been reported [147]. This observation contradicted the notion that vitamin D is a chemo-protective, or tumoristatic agent. In another study performed in Italy, vitamin D intake was observed to be beneficial with respect to its hypothetical influence on melanoma risk [148].

Brozyna *et al.* [149] reported that a decrease in expression of vitamin D receptors had occurred, as skin cancers had progressed. This was indicative that vitamin D activity, or its absence, had played some role in the progression of skin cancer. The anti-carcinogenic roles of vitamin D include decreasing proliferation and increasing differentiation of keratinocytes, taking part in DNA damage repair processes, and regulating the expression of oncogenic and tumor suppression of long non-coding RNAs (lncRNAs) in keratinocytes. With a reduced expression of Vitamin D receptors it follows that the anti-carcinogenic activities of vitamin D are also reduced [149]. Paradoxically, incidences of skin cancer have been associated with genetic damage and mutation within the skin, due to excessive exposure to the sun’s UVB rays, while concurrently, a relatively constant level of moderate sun exposure is believed to have protective effects against cancer, due to its role in the production of vitamin D [150].

#### 3.3.4. Vitamin E (Tocopherol)

Vitamin E is a fat soluble, essential nutrient, having anti-oxidant activities within the body [151]. Main dietary sources include vegetable oils and margarine, while other sources are nuts, seeds, egg yolks, asparagus, lettuce and whole grains [42,105]. Tocopherols (α, β, Δ and γ) are a group of compounds, which, together with the tocotrienols (α, β, Δ and γ), form the vitamin E family [152], with the tocotrienols exhibiting a more powerful anti-tumorigenic effect [153]. This section, however, mainly focuses on α-tocopherol, as it is the most abundant and active form of vitamin E.

Treatment of murine melanoma cells with α-tocopherol acid succinate was reported by Ottino and Duncan [154] to inhibit cell growth and cell proliferation. During their study, α-tocopherol acid succinate was proposed to exert the anti-proliferation effects via a COX pathway. The anti-cancer activity of α-tocopherol acid succinate occurred due to the α-tocopherol moiety and not because of the succinate salt, as had been indicated previously by Prasad and Edwards-Prasad [155]. Ottino and Duncan [156] determined in a separate study that vitamin E supplementation in murine melanoma cells resulted in an increase of cyclic adenosine monophosphate and adenylate cyclase activity, together with an elevation in prostaglandin E_2_ levels. These increases were related to the inhibition of cell growth [156]. Vitamin E succinate in a sesame oil vehicle was intra-peritoneally injected into tumor inoculated nude mice and it was observed that vitamin E had significantly inhibited melanoma tumor growth *in vivo* by inducing apoptosis [151]. Inhibition of melanoma growth and angiogenesis through the down regulation of VEGF by vitamin E succinate has also been observed in tumor inoculated mice [157].

A number of studies have been performed to determine the efficacy of vitamin E formulations as photo-protectants and chemo-protectants. Pedrelli *et al.* [158] found that a formulation, containing 10% of tocopherols and 0.3% of tocotrienols, had resulted in a photo-protective effect in humans, when topically applied prior to UV exposure [158]. Topical vitamin E has been reported to result in an increase in tumor burden in mice. Yet, in the same report a formulation with both vitamin C and vitamin E (CE Ferulic^®^) had resulted in a reduced tumor burden *in vivo*. It was suggested that vitamin E was effective in late stage tumorigenesis, as it was able to affect tumor progression and not initial tumor growth [159]. Possible utilization of nanoformulation in the development of vitamin E based formulations for cancer treatment, in combination with other anti-cancer agents, has been considered and investigated [160].

### 3.4. Terpenoids

Terpenoids are found in higher plants, mosses, liverworts, algae and lichens. These compounds are also referred to as terpenes or isoprenoids. Structurally, terpenoids are assembled from five carbon building units and the medically important sub-classes comprise the hemiterpenoids (C_5_), monoterpenoids (C_10_), sesquiterpenoids (C_15_) diterpenoids (C_20_), triterpenoids (C_30_), tetraterpenoids (C_40_) and polyterpenoids (C_5_)*_n_* [17]. Tetraterpenoids are also referred to as carotenoids (discussed in Section 3.2). Early uses of terpenoids included perfumes, flavorants, preservatives and pigments, but their applications have long since diversified to include medicines, soaps and narcotics [161]. A commercial anti-neoplastic agent has been developed from the diterpene, paclitaxel (Taxol), which was originally extracted from *Taxus brevifolia* [17].

*Ferula spp* and some mushroom (*Ganoderma lucidum* and *Coriolus versicolor*) are some of the plants containing terpenoid extracts that have been tested for cytotoxic activity against melanoma cells *in vitro*. A methanol extract and a purified methanol extract (mainly containing acidic terpenoids) of *Ganoderma lucidum* were prepared and used to determine their *in vitro* and *in vivo* anti-cancer properties. It was found that both extracts had shown strong anti-melanoma activity *in vitro* and they had reduced tumor volume *in vivo*, but the purified methanol extract was less potent than the methanol extract. The mechanisms by which these *Ganoderma lucidum* extracts exerted these activities were through oxidative stress, apoptosis induction and the inhibition of cell proliferation [162]. A terpenoid and polyphenol containing methanol extract of *Coriolus versicolor* was also tested for anti-melanoma activity *in vitro* and *in vivo*. The *Coriolus versicolor* methanol extract reduced melanoma cell growth and tumor volume through the inhibition of cell proliferation and the induction of apoptotic and necrotic cell death. This methanol extract also demonstrated synergistic effects on the anti-melanoma activities of macrophages [163]. The monoterpenes, *i.e.*, stylosin and tschimgine, were extracted from *Ferula ovina* and found to have potent cytotoxic activity against melanoma [164].

The photo-protective effect of the terpenoid β-damascenone was investigated *in vivo* and it was found that 20 µL of orally administered β-damascenone was able to protect UV light exposed mice from sunburn [165]. Tea tree oil is an example of a commercial product that contains varied terpenoids, and is extracted from *Melaleuca alternifolia.* Tea tree oil has been found to exhibit some anti-cancer activity and is relatively safe if taken in low concentrations, thus making it an ideal candidate for further investigation with respect to its anti-cancer potential [166]. Actions of home remedies of tea tree oil, as reported by the public, include being effective against actinic keratosis, basal cell carcinoma (BCC) and squamous cell carcinoma (SCC). Tea tree oil in 10% dimethylsulphoxide resulted in a direct cytotoxic effect on tumor cells and induced local immune activation when applied topically. Studies performed on subcutaneous mesothelioma in mice revealed that one day after topical application of tea tree oil, the tumor cells had shown signs of damage and death, such as compressed nuclei, contracted chromatin and swollen mitochondria. By day three the mitochondria had burst and the endoplasmic reticulum had dissolved. These results serve as an indication that topical application of tea tree oil could potentially be used in skin cancer therapy [167].

### 3.5. Resveratrol

**Figure 5:**
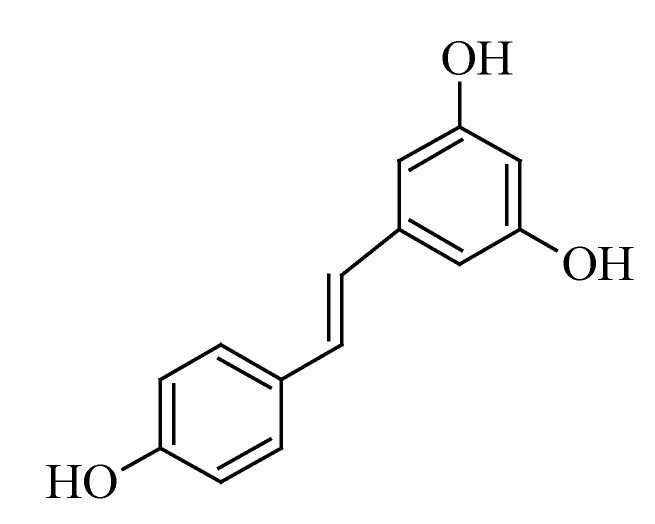
Chemical structure of resveratrol.

Resveratrol is a plant phytoalexin that is classified as a group A stilbene. Stilbenes are part of the stilbenoid family of compounds found in nature that are characterized by two aromatic rings, being joined by a methylene bridge [168]. Resveratrol (Figure 5) is a model stilbene and possesses a number of pharmacological activities, such as cardio-protection, chemo-prevention and anti-tumor activities [169]. The main source of resveratrol is grape vine skin, where it serves to protect the plant from bacterial (*Bothrytis cinerea*) infection. Other sources are Japanese knotweed (*Polygonum cuspidatum*) and Mojave yucca plant (*Yucca schidigera*). It is believed that resveratrol is capable of affecting the carcinogenesis process in the tumor initiation, -promotion and -progression phases [170].

Resveratrol has been investigated as an anti-cancer agent and it has been found that it is capable of inhibiting the growth of melanotic and amelanotic cells through apoptosis induction [171]. The potency of resveratrol has been demonstrated by its ability to induce apoptosis in doxorubicin resistant murine melanoma cells and its ability to inhibit growth of doxorubicin resistant melanoma tumors in mice [172]. Resveratrol possesses some anti-metastatic potential, as it has been reported to inhibit lipopolysaccharide induced epithelial to mesenchymal transition, possibly by inhibiting NF-κB signaling [173]. There is also potential for application of resveratrol as a radiation sensitizer in melanoma treatment, as it has been observed that radio resistant melanoma cells had responded well to a treatment combination of resveratrol and radiation [174]. The combined treatment showed better results, compared to treatment with either radiation, or resveratrol alone. A combination of resveratrol and temozolomide has been found to act as an effective cytotoxic agent against melanoma cells *in vitro*, however, the *in vitro* effects were not translatable into *in vivo* effects after intra-peritoneal administration [175]. This phenomenon was confirmed by the findings of Niles *et al.* [176], who reported that orally administered resveratrol had not inhibited melanoma tumor growth in mice [176]. It has been argued that the *in vivo* anti-cancer effects of resveratrol are strongly limited by its low bioavailability [177]. It seems that resveratrol works well in combination with other treatment modalities, as it has also been reported that resveratrol sensitizes melanoma cells to interleukin (IL) 2 immunotherapy induced cell death [178].

### 3.6. Curcumin

**Figure 6:**
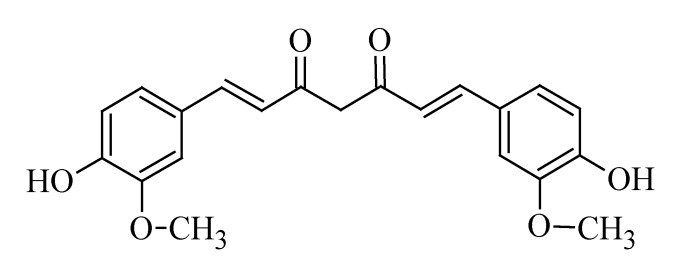
Chemical structure of curcumin.

Curcumin (Figure 6) is a yellow plant polyphenol that has been used over the years as a spice and as a medical agent. The main medicinal properties being attributed to curcumin are its anti-inflammatory and anti-oxidant activities. *Curcuma longa*, better known as turmeric, is the main source of curcumin. The curcumin being used in some studies usually contains a mixture of curcuminoids in which only 2%–6% comprise of curcumin. Commercial grade turmeric contains ~80% curcumin, which may account for any inconsistencies in outcomes, as observed among different studies [179]. Although curcumin has not been approved for the treatment of any disease, it has been established that curcumin exhibits some efficacy against various diseases (epilepsy, cancer, human immunodeficiency virus, diabetes and psoriasis), whilst also having a good safety profile at gram doses [180]. The possible application of curcumin as a drug for controlling and treating cancer symptoms, such as pain, depression, fatigue and neurodegeneration, has been investigated by various researchers and seemed promising [181].

In a recent study it was found that the anti-melanoma activity of curcumin was dependent upon the opening of mitochondrial permeability transition pore (mPTP), as had been accomplished by curcumin in melanoma cells *in vitro* [182]. Curcumin is able to induce apoptosis that is independent of p53 activity in melanoma cells in a time and dose dependent manner. Treatment of melanoma cells with curcumin resulted in inhibition of the NF-κB pro-survival pathway and activation of the death receptor Fas-initiated Fas-Associated protein with Death Domain (FADD) apoptotic pathway via caspase 8 [183]. Studies were performed to observe the effects of topically applied curcumin on UVB induced carcinogenesis in mice. It was observed that both pre- and post UVB exposure to topical applications of curcumin had resulted in a delay in tumor appearance, and a reduction in tumor multiplicity and tumor volume, without causing any toxic effects. Exposure to curcumin also resulted in an increase in the p53 tumor suppressor protein [184]. Oral administration of curcumin has been reported to cause down regulation of anti-apoptotic Bcl-2 and proliferating cell nuclear antigen (PCNA) in subcutaneous melanoma tumors, possibly regulated by microRNA [185]. Besides inhibiting growth of melanoma, curcumin inhibited growth of squamous cell carcinoma tumor through inhibition of ribosomal S6 phosphorylation [186].

Curcumin is a hydrophobic compound with low solubility and oral bioavailability, hence presenting challenges for use in the possible treatment of illnesses. Research is being carried out regarding the incorporation of curcumin into nanoformulations, such as liposomes, cyclyodextrins, solid dispersions and lipid nanoparticles, aimed at increasing its solubilization [187,188]. A comparison was made between the physicochemical properties of ethosomes, traditional liposomes and propylene glycol liposomes and their prospective uses as curcumin transdermal delivery vehicles. Propylene glycol liposomes showed favorable characteristics with respect to curcumin release profiles, particle size and encapsulation efficiency [189]. A microemulsion drug delivery system, comprising of limonene, has been reported to have promising properties for the transdermal delivery of curcumin. Successful formulation of a curcumin transdermal microemulsion should allow for the optimized delivery of curcumin to the skin where it would exert its anti-melanoma effects [190]. Moorthi and Kathiresan [191] have proposed the use of dual drug loaded (*i.e.*, curcumin-piperine, curcumin-quercetin, or curcumin-silibinin) nanoparticulate formulations in order to address the risk of multi-drug resistance formation, water insolubility and low bioavailability [191].

### 3.7. Sulforaphane

**Figure 7:**
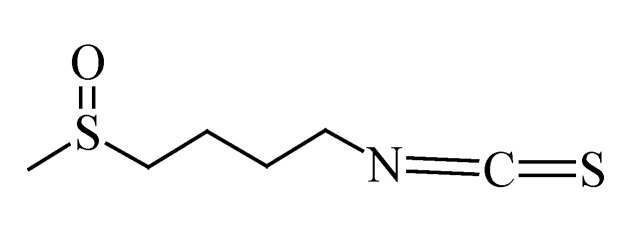
Chemical structure of sulforaphane.

Sulforaphane is an isothiocyanate found in cruciferous vegetables, such as broccoli (*Brassica oleracea* var*. italic*), cabbage (*Brassica oleracea* var. *capitata*), radish (*Raphanus sativus*), kale (*Brassica oleracea* var. *acephala*) and cauliflower (*Brassica oleracea* var. *botryitis*) [192]. The chemical structure of sulforaphane is shown in Figure 7. The anti-cancer activities of sulforaphane being reported include apoptosis induction, inhibition of cell proliferation and the inhibition of metastasis. Studies have been performed on melanoma cells to confirm whether sulforaphane would exhibit these activities in melanoma cells.

Sulforaphane, like other compounds, acts on various receptors and pathways in order to induce apoptosis in melanoma cells. Sulforaphane induces apoptosis by up regulating caspase 9, caspase 3, p53 protein and the Bax gene. It also down regulates Bcl-2, Bid and caspase 8, and thus inadvertently works against the apoptosis process [193,194]. *In vivo* studies on the actions of sulforaphane in a murine melanoma model have revealed that sulforaphane is anti-metastatic and has potential for application in cancer immunotherapy. Sulforaphane inhibits metastasis by stimulating the cell mediated immune response, by up regulating IL-2 and interferon gamma (IFN-γ), while down regulating IL-1β, IL-6, TNF-α and *granulocyte-macrophage colony-stimulating factor* (GM-CSF) [195,196].

Sulforaphane is reported to be unstable at temperature conditions between 60–90 °C, has a very short half-life and a low bioavailability, which makes it unsuitable for formulation into a clinically effective product [197,198]. Do *et al.* [198] investigated the potential of albumin microspheres as sulforaphane drug delivery agents in mice. Mice were subcutaneously injected with B16 melanoma cells to form tumors. The mice were then treated with sulforaphane microspheres through intra-peritoneal injection and it was observed that sulforaphane from the microspheres had a sustained inhibitory effect on tumor growth, compared to the sulforaphane solution [198]. In a later study, sulforaphane loaded magnetic microspheres were formulated. Compared to the albumin microspheres, the magnetic microsphere increased therapeutic efficiency by 10% [199].

## 4. Anti-melanoma Activity of Crude Plant Extracts

### 4.1. Hypericum perforatum

Hypericin, a penanthroperylene quinone and known for its photo-sensitizing effects, is one of the active components of St. John’s Wort (*Hypericum perforatum*). The concentration and light dose dependent photo-sensitizing effects of hypericin make it an ideal candidate for use in photodynamic therapies (PDT) of skin cancers. Davids, *et al.* [200] found that hypericin had been activated by UVA and resulted in melanoma cell death through the processes of apoptosis and necrosis [200,201]. However, the photo-sensitizing effects of clinically used aminolevulinic acid methyl ester have been found to be much superior to the effects of hypericin in photodynamic therapies [202]. This may have been due to its properties, such as low solubility and low stability in solution, which are relatively unfavorable for topical application [203]. A hydro-alcoholic extract of *H*. *perforatum* was tested against human malignant melanoma cells and it was observed to inhibit free radical formation, inhibit cell proliferation and to enhance UVA induced photo-toxicity [204]. According to Skalkos, *et al.* [203], a polar methanolic extract of *H. perforatum* has good physical and fluorescence properties, which make it an ideal candidate for further investigation for use as a PDT photosensitizer and thus for possible use in PDT of skin cancer [203].

### 4.2. Withania somnifera

Ashwaghanda or Indian ginseng (*Withania somnifera*) is a plant from the Solanaceae family and is the main source of a group of potent medicinal compounds, called withanolides. Two melanoma cell lines were treated with natural and derivative withanolides and an anti-proliferative effect was observed [205]. The most powerful and commonly studied withanolide is withaferin A (WA) and it is reported that WA could be useful in hyperthermia treatment of melanoma. Withaferin A resulted in an increased tumor response during hyperthermia treatment, whilst a decrease in the extent of thermo-tolerance was observed [206]. The combination of radiotherapy, hyperthermia treatment and a non-toxic dose of WA has been suggested as a possible alternative for melanoma therapy, following findings that such a combination had resulted in a better therapeutic outcome than radiation alone in a murine model [207]. Withaferin A alone induces apoptosis in melanoma cells by initiating production of ROS and by down regulation of Bcl-2 [208], while a methanolic extract of *Withania somnifera* has been found to inhibit metastasis in a murine melanoma model [209].

### 4.3. Melaleuca alternifolia

Tea tree oil, extracted from *Melaleuca alternifolia*, is well known for its various medicinal properties, especially with regards to the skin. The main active component of *M. alternifolia* is terpinen-4-ol and its anti-cancer activity has been researched. Terpinen-4-ol and tea tree oil have been reported to inhibit melanoma cell growth *in vitro* through cell cycle arrest, apoptosis, necrosis and inhibition of cell proliferation at doses that are not toxic to normal fibroblast cells [210]. The effect of topical application of 10% tea tree oil in dimethylsulphoxide to subcutaneous melanoma tumor bearing mice was also investigated and it was reported that a significant inhibition in tumor growth had been observed [211]. Generally, oil from *M. alternifolia* and its terpene components have proven to inhibit the *in vitro* and *in vivo* growth of melanoma cells and tumors [211,212].

### 4.4. Zingiber officinale

*Zingiber officinale* (ginger) is a monocotyledonous herb, which exists as a rhizome. An active component of *Zingiber officinale*, [6]-gingerol, has been investigated for its anti-cancer potential in melanoma and epidermoid carcinomas cells (SCC). [6]-Gingerol potently inhibited VEGF induced angiogenesis, but had no direct effect on the melanoma cells [213]. The effects of [6]-gingerol on epidermoid carcinoma cells have been reported to include growth inhibition, anti-proliferative and apoptosis induction. The apoptotic action seems to be regulated by ROS [214]. According to these reports, [6]-gingerol inhibits melanoma tumor growth by affecting the venous supply to the tumor, but it is capable of causing cell death through apoptosis in SCC.

### 4.5. Viscum album

*Viscum album* (*V. album*), well known as mistletoe, has been reported to improve the quality of life of cancer patients, and have shown some anti-cancer and immuno-stimulatory activities. Most studies have been performed on European mistletoe (*Viscum album loranthaceae*), but activities of Korean mistletoe (*Viscum album* *coloratum*) are also reported on. Lectins, isolated from Korean mistletoe, are reported to have prophylactic and therapeutic metastasis inhibitory actions. The anti-metastatic action is thought to occur by way of natural killer cell and macrophage activation [215,216]. Anti-angiogenic activity at the primary and metastatic sites is also responsible for the anti-metastatic effect of Korean mistletoe [217]. European mistletoe lectins directly act on human melanoma cells, causing dose dependent apoptosis [218]. These lectins were found to inhibit tumor growth in mice, due to the immuno-modulatory effects of interleukin-12 that enhance the functions of T-cells and NK-cells [219]. A terpene extract of European mistletoe mainly containing oleanolic- and betulinic acid is capable of inducing early stage apoptosis and late stage necrosis in melanoma cells [220]. In a comparative, epidemiological cohort study, the influence of subcutaneous injection (2–3 times/week) of *V. album* to patients suffering from stage II–III primary malignant melanoma, who had been surgically treated, was observed. Long-term treatment with *V. album* seemed to have significant survival benefits [221]. A case study of a patient suffering from metastatic malignant melanoma has shown that subcutaneous mistletoe therapy seems safe and could possibly result in a complete remission of metastasis [222].

### 4.6. Calendula officinalis

Extracts from the flowers of *Calendula officinalis* (*C. officinalis*) (marigold) are well known for their anti-inflammatory and anti-cancer properties. Jimenez-Medina *et al.* [223] reported that a laser activated extract of *C. officinalis* had inhibited cell growth in murine melanoma cells and had also inhibited tumor cell proliferation in human cells [223]. Some triterpene glycosides from *C. officinalis* extract exhibited potent cytotoxicity against melanoma and further studies on the individual compounds, or combinations thereof are recommended [224]. Marigold was reported to have an anti-cancer effect that is highly selective for human melanoma Fem-x cells, with 50% inhibitory concentration (IC_50_) values of 0.36 ± 0.12 mg/mL, compared to those for HeLa cells (0.75 ± 0.21 mg/mL) and for human colon carcinoma cells (2.30 ± 0.08 mg/mL). Marigold tea showed higher anti-melanoma action, compared to chamomile (*Matricaria chamomilla*) tea, with an IC_50_ value above 16.67 mg/mL [225]. Orally administered *C. officinalis* extract furthermore showed anti-metastatic effects that led to an increase in the life span of metastatic tumor bearing mice [226].

### 4.7. Rosmarinus officinalis

*Rosmarinus officinalis* (*R. officinalis*) is a herbal, evergreen plant, commonly referred to as rosemary. It contains phenolic diterpene and triterpene anti-oxidants [227]. Carnosol, a phenolic diterpene, is an active constituent of *R. officinalis* extract. In a study by Huang, *et al.* [228], carnosol had inhibited the migration of metastatic murine melanoma cells into a basement membrane gel and this effect was attributed to the inhibition of MMP-9. Carnosol was also reported to decrease cell viability and cell growth at high concentrations [228]. Another active constituent of *R. officinalis* is the pentacyclic triterpene, *i.e.*, ursolic acid. Ursolic acid has been observed to stimulate expression of the p53 protein and to inhibit activation of NF-κB in human and murine melanoma cells, which in turn resulted in apoptosis [229,230]. In another study, ursolic acid had enhanced apoptosis, induced by radiotherapy, thus indicating a possible role for it as an adjuvant to radiotherapy in the treatment of melanoma [231]. The radiation therapy potentiating effects of ursolic acid were established to have been partly caused by p53 activation through adenosine monophosphate activated protein kinase/mitogen activated protein kinase (AMPK/MAPK) signaling [232]. Additionally, ursolic acid has been classified as a potent anti-angiogenic agent in melanoma, due to its inhibitory effects on the production of VEGF, MMP-2, MMP-9 and nitric oxide [233].

### 4.8. Aloe Species

Aloe is a plant that is well-known for its medicinal properties and specifically for applications, such as wound healing, as a laxative and for the treatment of skin irritations [26]. Murine melanoma cells were treated with saline extracts of *Aloe vera* and was it observed that such treatments had reduced cell viability in a concentration dependent manner [234]. Emodin, an active component of Aloe, is a natural hydroxyanthraquinone, which has been studied for its possible anti-melanoma properties. The anti-melanoma effects of aloe-emodin included time dependent anti-proliferation and inhibition of MMP-9. Aloe-emodin treatment had resulted in the interference of murine melanoma cell aggregation, migration, invasion and adhesion, which presented as anti-metastatic activities [235]. During a study by Radovic, *et al.* [236], they determined that aloe-emodin had reduced the growth of human and murine melanoma cells and had promoted cell differentiation. Unexpectedly, in the presence of other toxic stimuli (*i.e.*, doxorubicin and paclitaxel), aloe-emodin had antagonized the cytotoxic actions of the toxic stimuli and was it cyto-protective instead [236]. Another compound found in Aloe, namely aloin, was reported to have significantly inhibited melanoma cell growth, interfered with cell adhesion processes and had sensitized melanoma to the cytotoxic agent, cisplatin [237].

### 4.9. Artemisia Species

Artemisia is a plant genus consisting of over 500 different species of aromatic herbs or shrubs. Eupatilin, a flavonoid that is isolated from *Artemisia* species, has shown to inhibit cell growth, induce apoptosis and induce G2/M cell cycle arrest in human melanoma cells [238]. Other previously studied artemisin derived compounds are dehydroleucodine, dehydroparishin-B from *A. douglasiana* (California Mugwort) and ludartin from *A. amygdalina* (almond wormwood). Dehydroleucodine and dehydroparishin-B have been found to block cell proliferation and inhibit murine melanoma cell migration [239]. Ludartin is also reported to have cytotoxic activity against human epidermoid carcinoma (IC_50_ of 19.0 µM) and mouse melanoma cells (IC_50_ of 6.6 µM) [240]. Besides individual compounds, *Artemisia* essential oils have also been tested for cytotoxic activity. Essential oils extracted from *A. anomala* (diverse wormwood herb) were tested for cytotoxic activity against melanoma and were reported to have an IC_50_ value of 0.2 µL of oil per mL [241].

### 4.10. Alpinia Species

Extracts from various *Alpinia* species have been tested for possible anti-cancer activities. Extracts from *A. oxyphylla* (sharp leaf galangal), *A. galangal* (greater galangal) and *A. officinarum* (lesser galangal) have in particular been tested for possible anti-melanoma activities. An extract of *A. oxyphylla* from supercritical fluid carbon dioxide was found to act as an anti-proliferation agent in human melanoma cells [242]. Two compounds extracted from *A. galangal*, *i.e.*, 1,7-bis(4-hydroxyphenyl)-1,4,6-heptatrien-3-one and bisdemethoxycurcumin, are reported to have significantly inhibited melanoma cell proliferation [243]. Galangin, a compound from *A. officinarum*, had suppressed cell proliferation and induced apoptosis via the mitochondrial pathway and through activation of p38 MAPK [244]. In another study it was further determined that galangin had inhibited cell adhesion, spreading, motility and lamellipodia formation *in vitro. In vivo*, galangin was reported to have inhibited lung metastasis in a mouse melanoma model [245].

## 5. Conclusions

From this review, is has become clear that naturally derived compounds could very likely become key role players in future melanoma treatments. This article has summarized some of the compounds and plants that have been studied to date for their possible anti-cancer properties. Many more untapped resources, however, remain in nature. Phytochemicals have been reported to possess numerous health benefits and ongoing research is conducted to determine their physiological effects. Anti-cancer activities of plants can be ascribed to a distinct compound, or to a combination of the effects of different compounds in the crude extract and/or in the human body. Although all of the compounds and crude extracts, discussed in this review, are reported to have demonstrated some form of anti-cancer activity *in vitro*, most of these identified actions must still be clinically proven for indeed causing favorable clinical effects in humans.

Traditional use of natural compounds in cancer treatment is relatively cheap due to the availability of plants and the simple methods used in product preparation. However, commercialization of natural compounds for cancer treatment may result in dwindling of natural resources and problems with producing a consistent quality of adulteration. Due to this, most naturally derived medicinal compounds are eventually manufactured semi-synthetically, through *in vitro* cultivation or fermentation technology for commercial use then formulated into an appropriate dosage form which increases costs. Similar to conventional drugs herbal medicinal products can be toxic if they accumulate beyond the acceptable level in the human body. Another safety issue which exists is misidentification of a plant coupled with unavailability of analytical methods to confirm plant species which may lead to adverse effects [17]. There are various advantages to traditional use of herbal remedies but it is vital for toxicology data to be available in order to avoid toxicity issues. Scientific research is needed in order to optimize the herbal medicinal products for safe human use.

Since some natural products have shown the potential for use in the symptomatic treatment of cancer, or to treat the adverse effects associated with cancer therapies, this has led to an increase in self-medication by cancer patients, seeking safer and more effective products. In certain cultures, traditional healers have been reported to formulate herbal mixtures of unpurified and non-quantified extracts, which are given as tinctures for the treatment of diseases, such as cancer, without any scientific evidence of the efficacy and safety of these natural medicines. A need for sound scientific research to bridge the gap between certain medicines from natural origin and conventional prescription medicines therefore exists. Patients often associate natural products with safety, but many herbal or natural products are not necessarily safe, as they can cause adverse effects, either alone, or due to an interaction with other substances/drugs in the body. Practitioners must therefore be educated in the science of herbal medicines, so that they can appropriately advise their patients regarding the safe and suitable use of complementary and alternative medicines [246,247].

Assessing the quality of herbal or supplemental therapies offered by complementary and alternative medicines is quite a challenge, as some therapies are individually tailored. Concern also exists with respect to the concentrations used and the standardization of formulations, as most natural products available on the market are not classified as medicines and are thus not regulated by any monitoring body. Due to the lack of regulation, large variations in formulations and bioactivity among batches are expected, since the active components, minimum effective concentrations and maximum safe concentrations are usually unknown [248]. Healthcare professionals are advised to practice pharmaco-vigilance in order to detect possible adverse interactions between prescription medication and non-conventional medicine. It is also suggested that large, randomized trials should be conducted that would assist in determining the effectiveness of herbal medicines [249]. The US National Institute of Health (NIH), National Centre for Complementary and Alternative Medicine (NCCAM) is making strides to determine the safety and effectiveness of various CAM botanical therapies through funding of researchers [250]. There has been an increase in research on the safety and effectiveness of CAM and the expectation is that an increase in the availability of accurate information on the web will result in informed decisions and good outcomes for patients interested in CAM [251,252,253]. In light of this, online news sites have been urged to ensure that they report accurate news with respect to CAM, as many people seek health advice on the internet [254].

## Abbreviations

AMPK: adenosine monophosphate activated protein kinase
Bax: Bcl-2 associated X protein
BCC: basal cell carcinoma
Bcl-2: B cell lymphoma 2
CAM: complementary and alternative medicine
CDK: cyclin dependent kinase
Cip: CDK interacting protein
CMM: cutaneous malignant melanoma
COX-2: cyclooxygenase 2
CXCR4: C-X-C receptor 4
DNA: deoxyribonucleic acid
EGCG: epigallocatechin-3-gallate
ERK: extracellular signal-regulated kinase
FADD: Fas-Associated protein with Death Domain
FDA: Food and Drug Administration
GM-CSF: *granulocyte-macrophage colony-stimulating factor*
*IC_50_*: *50% inhibitory concentration*
IFN: interferon
IGF-1R: Type 1 insulin like growth factor receptor
IL: interleukin
lncRNA: long non-coding RNA
MAPK: mitogen activated protein kinase
MMP: matrix metalloproteinase
mPTP: mitochondrial permeability transition pore
mRNA: m-ribonucleic acid
NCCAM: National Centre for Complementary and Alternative Medicine
NCI: National Cancer Institute
NF-κB: nuclear factor kappa-light-chain-enhancer of activated B cells
NIH: National Institute of Health
PCNA: proliferating cell nuclear antigen
PDT: photodynamic therapy
PI3K: phosphoinositide-3 kinase
RAR: retinoic acid receptor
ROS: reactive oxygen species
SCC: squamous cell carcinoma
STAT-3: *signal transducer and activator of transcription 3*
TNF-R: tumor necrosis factor receptor
TRAIL-R: TNF-related apoptosis inducing ligand receptor
USA: United States of America
UV: ultra-violet
VEGF: vascular endothelial growth factor
